# A prognostic model of lung adenocarcinoma constructed based on circadian rhythm genes and its potential clinical significance

**DOI:** 10.3389/fonc.2025.1464578

**Published:** 2025-02-18

**Authors:** Cong Fu, Lin Sun, Cuncheng Feng, Tong Zhou, Yanzhi Bi

**Affiliations:** ^1^ Department of Oncology, Changzhou Cancer (Fourth People’s) Hospital, Changzhou, China; ^2^ Department of Oncology, Affiliated Hospital of Soochow University, Changzhou, China; ^3^ Department of Gastrointestinal Surgery, Affiliated Hospital of Nanjing Medical University, Changzhou No. 2 People’s Hospital, Changzhou, China

**Keywords:** risk model, lung adenocarcinoma, circadian rhythm, immune infiltration, bioinformatics analysis

## Abstract

**Background:**

Lung adenocarcinoma (LUAD) is a common pathological category of lung cancer. Circadian rhythm (CR) disruption has been demonstrated to impact on lung tumorigenesis in mouse models. The aim of this study was to mine genes relevant to CR in LUAD and construct a corresponding risk model.

**Methods:**

CRRGs from GSEA-MsigDB were filtered by overlapping DEGs in LUAD and NC specimens, two clusters with survival and clinical discrepancies, and CRRGs. Cox regression analysis (univariate and multivariate) was used to establish a CR-relevant risk model, which was validated in both the training and validation sets. Differences in immune infiltration, immunotherapy, and drug sensitivity between subgroups were explored. Prognostic gene expression was tested in clinical cancer and paracancer tissue samples using RT-qPCR.

**Results:**

A grand total of two prognostic genes (*CDK1* and *HLA-DMA*) related to CR were screened. The AUC values of a CR-relevant risk model in predicting 1/3/5-years survival in LUAD patients were greater than 0.6, indicating that the efficiency of the model was decent. Then, the results of CIBERSORT demonstrated noticeable differences in the tumor microenvironment between CR-relevant high- and low-risk subgroups. In addition, the CR-relevant risk score could be performed to estimate the effectiveness of immunotherapy in LUAD patients. The sensitivity of three common drugs (homoharringtonine, lapatinib, and palbociclib) in LUAD could be evaluated by the CR-relevant risk model. Ultimately, the experimental results confirmed that the expression trends of *CDK1* and *HLA-DMA* in our collected clinical samples were in line with the expression trends in the TCGA-LUAD dataset.

**Conclusion:**

In conclusion, a CR-relevant risk model based on *CDK1* and *HLA-DMA* was constructed by using bioinformatics analysis, which might supply a new insight into the improved prognosis of LUAD.

## 1 Introduction

Lung cancer is a highly prevalent cancer worldwide and seriously affects human health and life safety (1). It is the most common malignancy in men with the highest morbidity and mortality and is the third most common in women with the second highest mortality (2). Lung adenocarcinoma (LUAD) is the most common subtype in non-small cell lung cancer (NSCLC), which accounts for about 40% of lung cancer cases (3). Due to the high aggressiveness of lung cancer, most lung cancer patients are diagnosed at an advanced stage, leading to a low 5-year survival rate of less than 20% in the majority of countries (4). Although the great development of targeted therapy and immunotherapy has improved the overall survival rate to a certain extent, lung cancer still has a poor prognosis. Therefore, finding reliable prognostic biomarkers is crucial to improving the treatment effectiveness and life quality of LUAD patients.

Circadian rhythm (CR) is a fundamental biological phenomenon wherein most physiological events in living organisms fluctuate regularly over a period of approximately 24 h. This fluctuation helps organisms adapt to the surrounding environment (5). Circadian clocks include the central clock located in the hypothalamic suprachiasmatic nucleus and peripheral clocks in various tissues throughout the body (6). The suprachiasmatic nucleus, as a central pacemaker, integrates light signals from the retina to regulate its own rhythm and synchronizes the peripheral clocks through various pathways involving mainly the autonomic nervous system and endocrine signals (7). At the molecular level, CR is generated by transcription–translation feedback loops, which are formed by a series of circadian clock genes and their protein products, e.g., CLOCK, BMAL, Cry, Per (8). CR regulates a wide variety of biological processes, including cell proliferation and differentiation, the cell cycle, DNA damage and repair, the immune response, and apoptosis (9). It also plays an important role in maintaining homeostasis and solid organ function.

The dysregulation of CR may contribute to a wide range of human diseases, including metabolic diseases (10), cardiovascular diseases (11, 12), sleep disorders, neurodegeneration (13), and especially cancer (14–16). CR dysfunction has been classified as a possible human carcinogen by the International Agency for Research on Cancer (IARC), a body of the World Health Organization (WHO), in 2007 (17). Accumulating epidemiological studies have shown that people who work night shifts are more susceptible to breast cancer (18), endometrial cancer (19), and prostate cancer (20). Two comprehensive analyses conclude that circadian pathway genetic variation is involved in cancer predisposition (21, 22). Regarding lung cancer, Gery et al. previously reported that the expression of Per1 is low in NSCLC patient samples and cell lines may be caused by DNA hypermethylation and histone H3 acetylation (23). Liu et al. reported that patients with a lower expression of Per1, Per2, and Per3 had shorter survival times and the loss of Per may promote tumor progression in NSCLC (24). A study using a genetically engineered mouse model has proved that genetic loss of Per2 or Bmal1, which play cell-autonomous tumor-suppressive roles in transformation and lung tumor progression, leads to increased c-Myc expression and promotes lung tumorigenesis (25). These results indicate that the abnormal expression of circadian rhythm genes may serve as novel prognostic biomarkers for LUAD.

Therefore, this study obtained LUAD-related data from The Cancer Genome Atlas (TCGA) database. Then, we screened the prognostic genes associated with CR by differential analysis, cluster analysis, and multiple regression methods and tried to establish a good risk model that could predict LUAD. Moreover, we explored the role of prognostic genes and the immune microenvironment of LUAD as well as the related drug sensitivity analysis. Eventually, the expression of prognostic genes was examined through clinical trials. Overall, the identification of prognostic genes in LUAD through these analyses lays a theoretical foundation for the diagnosis and treatment of the disease.

## 2 Materials and methods

### 2.1 Ethics approval and consent to participate

This retrospective study was approved by the institutional review board from Changzhou Cancer Hospital (2024(SR)NO.014).

### 2.2 Data source

We integrated clinical information and transcriptomic data of 535 lung adenocarcinoma (LUAD) specimens and 59 normal control (NC) specimens through the TCGA database. Then, 479 LUAD specimens with survival information were utilized to construct the risk model. The GSE30219 dataset (external validation set) including 115 LUAD specimens (lung tumor) was mined from the GEO database. Then, 300 CRRGs were extracted from the GSEA-MsigDB database with “rhythm” (26) (**Supplementary Table S1**).

### 2.3 Clustering analysis of LUAD specimens

To explore the relationship between circadian gene expression patterns and LUAD, consensus clustering was performed with the ConsensusClusterPlus package (v1.54.0) based on the expression data of CRRG in TCGA-LUAD samples (27). The clustering parameter was set to maxK = 6. Then, the points with the largest change in the cumulative distribution function (CDF) value were combined. Considering that a more stable CDF decreasing trend indicates better clustering, the best clustering method was selected. Subsequently, survival discrepancy between LUAD subgroups was delved via survival package (v3.2-11) to investigate the potential prognostic value of circadian-rhythm-related genes in LUAD (28). In addition, the discrepancy in clinical traits between LUAD subgroups was examined using chi-square test.

### 2.4 Differential expression analysis in TCGA-LUAD specimens

In order to identify genes with significant differences in expression levels between different sample groups, the limma package (v3.46.0) was implemented to identify the DEGs in LUAD and NC specimens (adj-*p* < 0.05, |log_2_ FoldChange (FC)| ≥ 0.5) (29). Then, the same criteria was utilized to extract DEGs between different clusters, and a volcano plot was generated using ggplot2 (v3.3.2) (30) to visualize the differential gene expression. Subsequently, CR-related DEGs were filtered by overlapping DEGs between LUAD and NC specimens, DEGs between different clusters, and CRRGs using the VennDiagram package (v1.6.20) (31).

### 2.5 Establishment of a CR-relevant risk model in LUAD

To investigate whether circadian genes are associated with the prognosis of lung adenocarcinoma patients, a prognostic model was constructed to predict the survival of lung adenocarcinoma patients. The LUAD specimens in the TCGA-LUAD database were randomly classified into training set (*N* = 336) and internal validation set (*N* = 143) in the ratio of 7:3. Firstly, the clinical expression data of LUAD was retrieved by merging overall survival (OS) information with the expression data of CR-related DEGs. Next, we used the “survival” package (v3.2-11) to perform Cox regression analysis (univariate and multivariate) on the training set (28). Multivariate Cox analysis was performed on the circadian rhythm genes with *p*-values <0.05 in the univariate Cox analysis. The genes were filtered using the step function based on AIC values to construct a risk model for lung adenocarcinoma. Depending on the median value of the CR-relevant risk score acquired by the following formula, LUAD patients were separated into two risk subgroups (high risk and low risk). $Riskscore=\sum_{i=1}^{n}coef(gene_{i})*\exp r(gene_{i})$ . The Kaplan–Meier (K–M) curve was plotted by using survminer package (v0.4.8) (32). For the purpose of assessing the effectiveness of the CR-relevant risk model, the survivalROC package (v1.0.3) was used to paint the ROC curve with 1, 3, and 5 years as the survival time node (33). Finally, the CR-relevant risk model was verified in both the internal validation set and the GSE30219 dataset.

### 2.6 Analysis of clinical parameters and nomogram creation

To further investigate the prognosis of the clinical pathological features and risk models, a correlation analysis was conducted between clinical factors and risk scores in the LUAD training set samples. Firstly, a chi-square test was utilized to compare the number of patients with various clinical parameters (vital, stage, TMN stage, gender, smoking category, and age) between two risk subgroups. Then, the discrepancy in risk score between two subgroups was assessed via Wilcoxon test for the abovementioned clinical subtypes. Next, the survival discrepancies between the clinical subtypes of patients in the high- and low-risk groups were compared. Subsequently, the abovementioned clinical parameters and risk score were imported in Cox regression analysis (univariate and multivariate) to authenticate independent prognostic factors. The nomogram was established via rms package (v6.2-10) to further investigate the prognosis of the risk model, and a dynamic column chart was created using the shiny package (https://jasmineonly.shinyapps.io/DynNomapp/) (34). Finally, a calibration curve was plotted to evaluate the predictive value of the nomogram.

### 2.7 Functional annotation for CR-relevant risk subgroups

In order to explore the functional differences between high- and low-risk groups, the enrichment scores of each KEGG pathway in specimens for the two CR-relevant risk subgroups were calculated by using the GSVA package (v1.36.3) (35). Then, pathways with significant discrepancies were screened using limma package (*p* < 0.01) (29). Finally, relevance analysis was conducted between differential pathways and risk scores depending on |cor| > 0.6 and *p* < 0.01.

### 2.8 Relevance analysis of CR-relevant risk model and immune infiltration

In order to investigate the differences in immune infiltration between high- and low-risk groups, the CIBERSORT algorithm (v1.03) was used to calculate the proportion of 22 immune-infiltrating cells in the training set samples (36), and samples with *P* > 0.05 were excluded. Subsequently, a correlation analysis was conducted on 22 types of immune cells, and a correlation heatmap was drawn. In addition, discrepancies in the 22 immune-infiltrating cells between two risk subgroups were compared by using Wilcoxon test (36). The box plot was plotted via ggplot2 (v3.3.2) (37) and Ggpubr package (v0.4.0) (https://CRAN.R-project.org/package=ggpubr).

### 2.9 Estimation of immunotherapy response

The expression and survival probability of 11 immune checkpoint genes (ICGs) extracted from the TCGA-LUAD were compared between two CR-relevant subgroups through Wilcoxon test and survival package. Then, a relevance analysis of ICGs and risk score was implemented via PerformanceAnalytics package (v2.0.4) (https://CRAN.R-project.org/package=PerformanceAnalytics). Wilcoxon test was utilized to examine the discrepancy of tumor mutational burden (TMB) obtained from cBioportal (https://www.cbioportal.org/) and TIDE score retrieved from TIDE database (http://tide.dfci.harvard.edu/) between the two subgroups.

### 2.10 Sensitivity analysis of chemotherapy drugs

The gene expression data of 60 cancer cell lines mandated by NCI and inhibitory concentration (IC50) of drugs approved by FDA were collected from the CellMiner database (https://discover.nci.nih.gov/cellminer/loadDownload.do). Relevance analysis of drugs and cell-line risk score calculated depending on the risk coefficients and expression data of model genes was implemented. Next, according to the median risk score value, cell lines were stratified into high- and low-risk groups, and the IC50s of drugs in the two groups were compared.

### 2.11 Analysis of expression for prognostic genes

Next, in order to verify the expression levels of genes identified from public datasets in clinical samples, 10 pairs of cancer and paracancerous tissue samples were collected from Changzhou Cancer Hospital. The tumor specimens were reviewed by two experienced oncopathologists. These were approved by the Changzhou Cancer Hospital ethics committee. All patients signed an informed consent form. The 50-mg samples from the abovementioned 10 pairs of tissues were lysed by adding TRIzol reagent (Ambion, USA) and homogenizing fully to obtain total RNA. Then, equal amounts of mRNA were reverse-transcribed into cDNA by using SureScript-First-strand-cDNA-synthesis-kit (Servicebio, China). After that, qPCR was implemented via 2xUniversal Blue SYBR Green qPCR Master Mix (Servicebio, China) and CFX Connect real-time quantitative PCR instrument (BIO-RAD, USA). The sequences of the qPCR primers for each gene and GAPDH are listed in **Supplementary Table S2**. The expression of prognostic genes was normalized using the conventional method with GAPDH as an internal reference gene (38).

### 2.12 Statistical analysis

All open databases and R software were utilized to analyze and visualize in this study. The heatmap was painted using pheatmap package (v0.7.7) (39). *P <*0.05 was taken as a significant difference.

## 3 Results

### 3.1 Subgroup analysis of LUAD specimens

**Figure 1:**
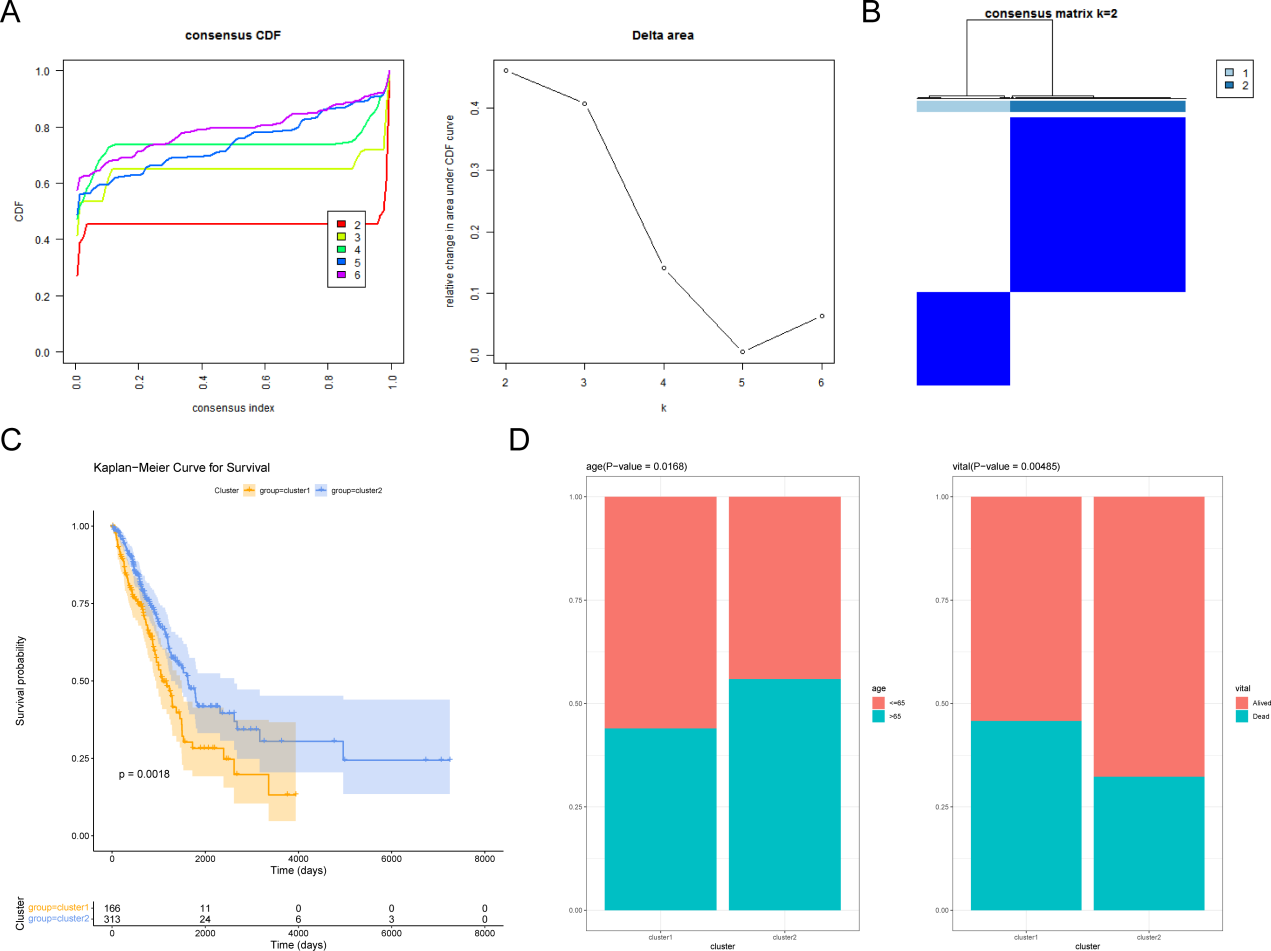
Exploring circadian rhythm (CR)-related genes in LUAD. **(A)** Consistency clustering cumulative distribution function (CDF) plot. The horizontal axis represents the consistency index, ranging from 0 to 1, with different colors indicating different numbers of classes. **(B)** Sample clustering heatmap. The darker the blue, the easier it is to cluster together. **(C)** Survival curves of patients in cluster 1 and cluster 2. **(D)** Differences in age and vital subtypes between cluster 1 and cluster 2.

On the basis of 300 CRRGs, the LUAD specimens were classified into two clusters (*k* = 2) (**Figures 1A, B**). The K–M curve indicated a significant difference in survival between cluster 1 and cluster 2, with cluster 1 having a lower survival probability (*P* = 0.0018) (**Figure 1C**). In addition, a clinical differential analysis showed significant differences in age (>65 or ≤65 years) and vita status (alive or dead) between the two clusters (**Figure 1D**; **Supplementary Figure S1**). In summary, CRRG could classify LUAD patients into two subtypes with differences in survival and clinical outcomes.

### 3.2 Identification of CR-related DEGs and construction of a risk model

**Figure 2:**
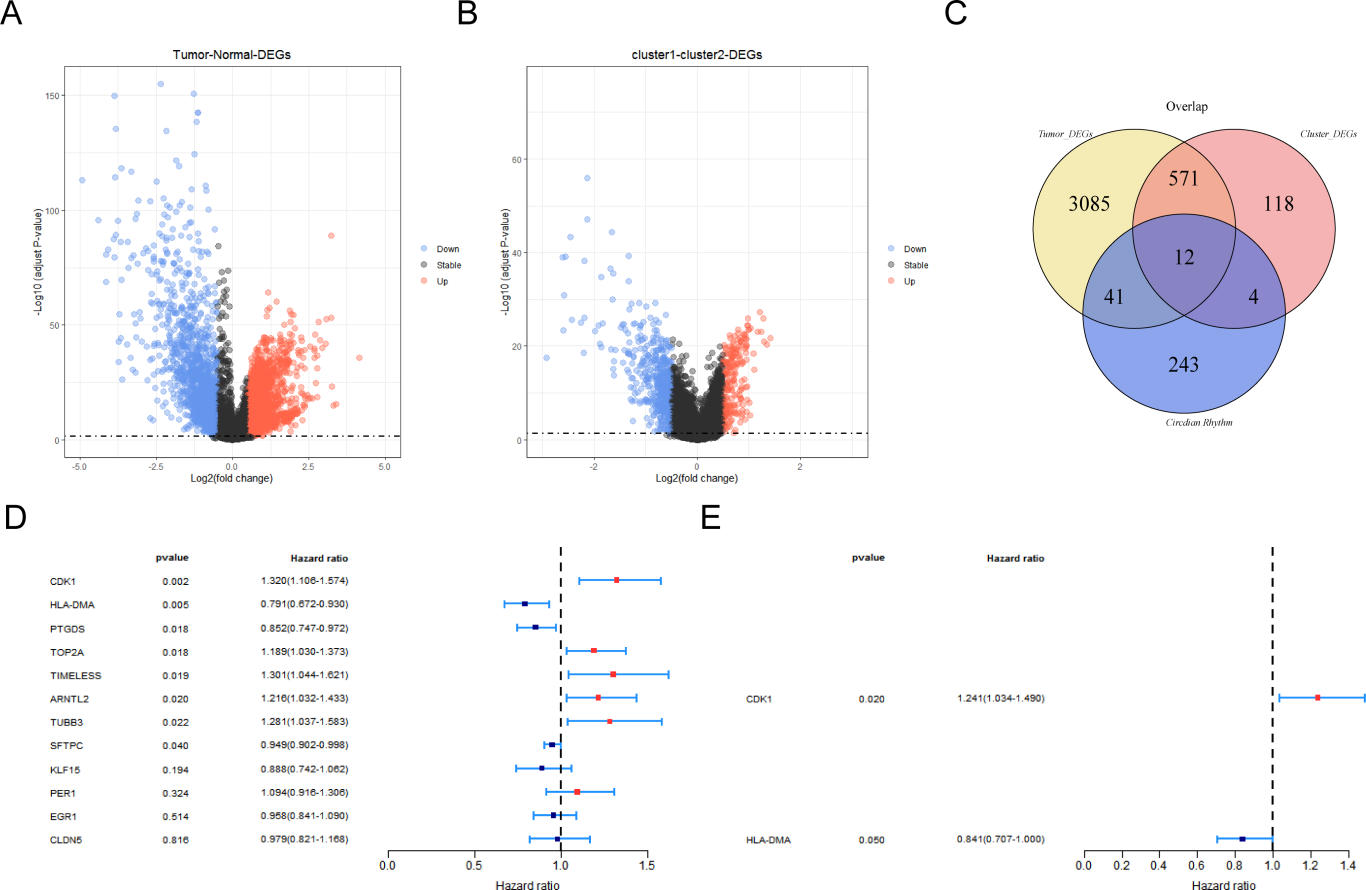
Screening of prognostic genes by regression analysis. **(A)** Volcano map of differentially expressed genes (DEGs) between LUAD and control groups. **(B)** Volcano map of differentially expressed genes among different clustering groups. Red indicates upregulated genes and blue indicates downregulated genes. Black indicates non-significant genes. **(C)** Venn diagram of circadian-rhythm-related DEGs. **(D)** Forest map for screening survival-related genes by univariate Cox regression analysis. **(E)** Forest map for screening prognostic gene by univariate Cox regression analysis.

A total of 3,709 DEGs were altogether recognized between LUAD and NC specimens in the TCGA-LUAD database, with 1,546 downregulated and 2,163 upregulated genes in LUAD specimens (**Figure 2A**; **Supplementary Table S3**). Moreover, 705 DEGs were recognized between cluster 1 and cluster 2, with 493 downregulated and 212 upregulated genes in cluster 1 specimens (**Figure 2B**). Then, 12 CR-related DEGs were obtained depending on the Venn diagram (**Figure 2C**; **Supplementary Table S4**).

The 336 LUAD patients with survival information in TCGA-LUAD served as the training set, while the remaining 143 patients served as the internal validation set. Firstly, eight survival-associated CR-related DEGs (CDK1, HLA-DMA, PTGDS, TOP2A, TIMELESS, ARNTL2, TUBB3, and SFTPC) were derived via univariate Cox analysis (**Figure 2D**). Subsequently, CDK1 and HLA-DMA were further screened out to establish a CR-relevant risk model by multivariate Cox analysis, with CDK1 being a risk factor (hazard ratio (HR) > 1) and HLA-DMA being a protective factor (HR < 1) for LUAD prognosis (**Figure 2E**; **Supplementary Table S5**).

### 3.3 CR-relevant risk model to assess the prognosis of LUAD patients

**Figure 3:**
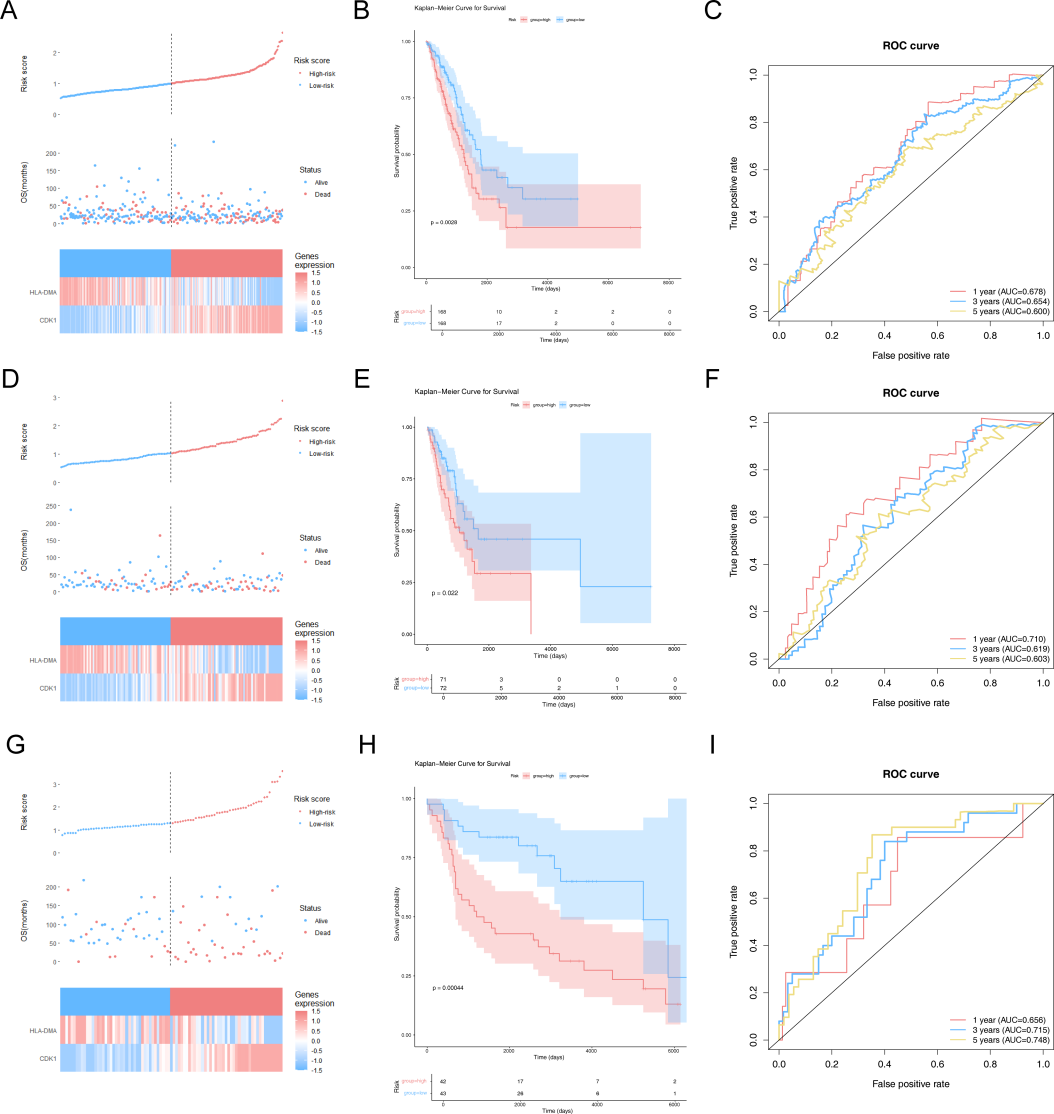
Construction and assessment of risk models. **(A)** Risk curves, scatter plots, and model gene expression heatmaps for high- and low-risk groups in the training set. The horizontal axis represents the patient samples sorted according to their risk scores. The vertical axis of the figure above represents the risk score, the vertical axis of the middle figure represents the survival time, and the following figure shows the expression heatmap of model genes for high- and low-risk groups. **(B)** Kaplan–Meier (K–M) survival curves in patients of high- and low-risk groups in the training set. **(C)** Receiver operating characteristic (ROC) curves at 1, 3, and 5 years for LUAD patients in the training set. **(D)** Risk curves, scatter plots, and model gene expression heatmaps for high- and low-risk groups in the internal validation set. **(E)** K–M survival curves in patients of high- and low-risk groups in the internal validation set. **(F)** ROC curves at 1, 3, and 5 years for LUAD patients in the internal validation set. **(G)** Risk curves, scatter plots, and model gene expression heatmaps for high- and low-risk groups in the GSE30219 dataset. **(H)** K–M survival curves in patients of high- and low-risk groups in the GSE30219 dataset. **(I)** ROC curves at 1, 3, and 5 years for LUAD patients in the GSE30219 dataset.

The LUAD patients were stratified into high- and low-risk groups depending on the risk score (median) for further evaluation of the prognostic value of the risk model (**Figure 3A**). A survival status analysis revealed increased odds of patient death as the risk score increases (**Figure 3A**). Moreover, the expression heatmap manifested that CDK1 was highly expressed in patients with a higher risk score, while HLA-DMA was highly expressed in patients with a lower risk score (**Figure 3A**). **Figure 3B** shows that the high-risk group was accompanied by a lower survival probability. In addition, the AUC values of 1/3/5 years for LUAD were all greater than 0.6, indicating a relatively accurate result (**Figure 3C**). To further demonstrate the reliability and usefulness of the risk model, the abovementioned analysis was also carried out both in the internal validation set and the GSE30219 dataset. We obtained almost identical results to the training set (**Figures 3D–I**). The results altogether indicated that the CR-relevant risk model was a valid survival predictor for LUAD patients.

### 3.4 Relevance analysis of clinical parameters and CR-relevant risk model

**Figure 4:**
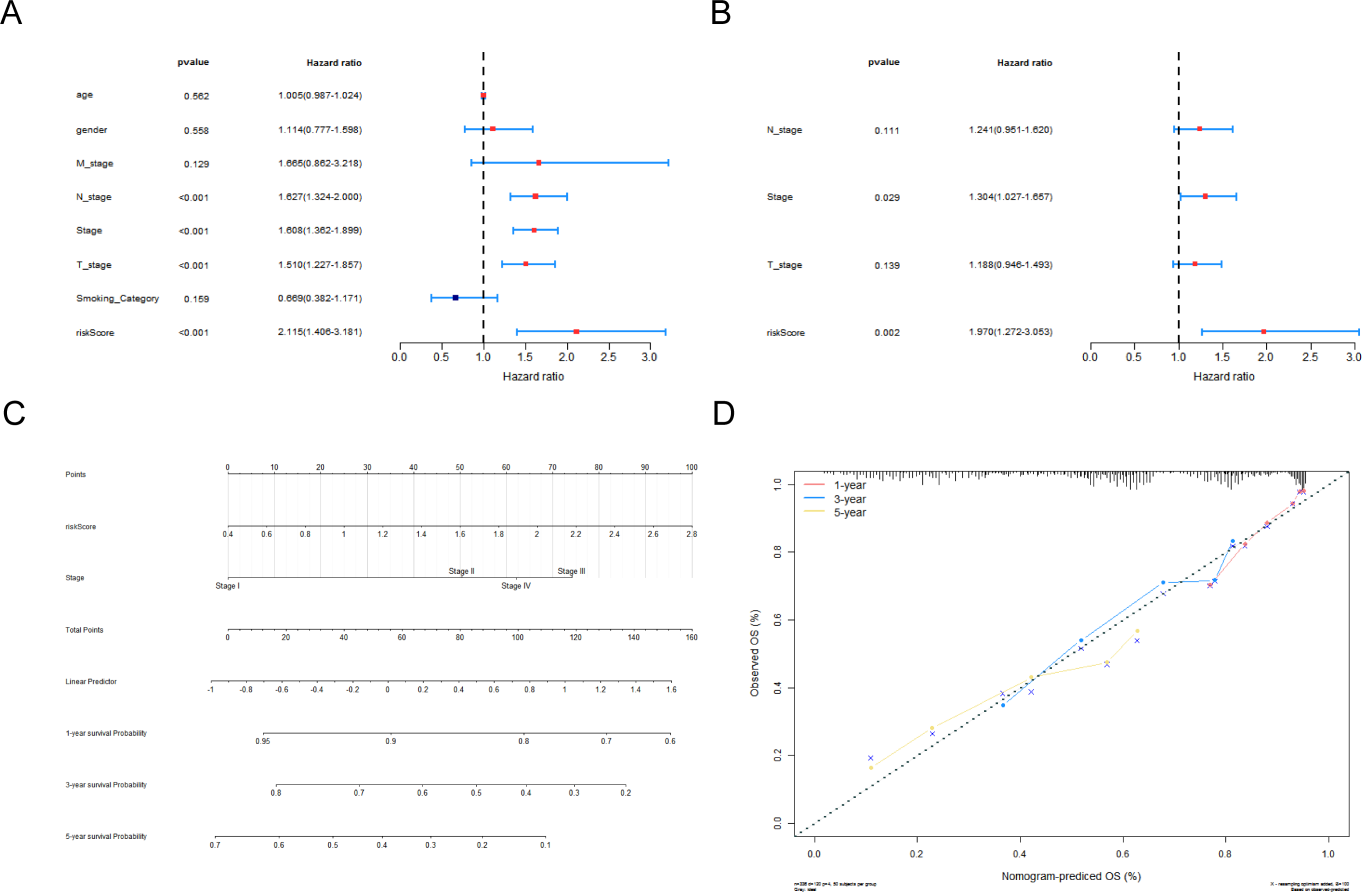
Construction and assessment of the nomogram of independent prognostic factors. Forest plots for univariate **(A)** and multivariate **(B)** Cox regression analyses. **(C)** Nomogram of independent prognostic factors that assessed the 1-, 3-, and 5-year survival probability in LUAD patients. **(D)** Calibration curves for nomogram.

To research the association between the risk model and the clinical characteristics of LUAD patients, we performed a relevance analysis. Firstly, the patient number of various clinical parameters had no discrepancy between the two risk subgroups except for vital status, stage, and T_stage (**Supplementary Table S6**). Then, the risk scores between the different clinical subtypes of vital status (dead or alive), stage (stage I + stage II or stage III + stage IV), age (≤65 or >65), gender (male or female), and N_stage (N0 or N+) had noticeable discrepancies (**Supplementary Figure S2A**). In addition, the survival discrepancies were presented in male, T1 + T2, M0, age >65, and non-smoker between two CR-relevant risk subgroups (**Supplementary Figure S2B**). To investigate the relevance between clinical parameters and the prognosis of LUAD, we excavated independent predictors via Cox regression analysis (univariate and multivariate). Hence, risk score and stage were reliable independent predictors for LUAD (**Figures 4A, B**). A nomogram (C-index = 0.7005) was established to predict the survival rate of LUAD patients depending on stage and risk score (**Figure 4C**). Finally, the calibration curve demonstrated the effectiveness of the nomogram (**Figure 4D**).

### 3.5 Risk-model-based study of the molecular mechanisms of LUAD

For the purpose of exploring the potential functions and pathways for the two risk subgroups, we implemented GSVA enrichment analysis. A total of 228 differential pathways were altogether authenticated, 11 of which were significantly associated with CR-relevant risk score (**Supplementary Tables S7**, **S8**). **Supplementary Figure S3A** reveals that all of the pathways, except the lysosome, were positively correlated with risk scores. In addition, the heatmap displayed cell growth and development, and DNA repair-relevant pathways were highly expressed in the high-risk subgroup (**Supplementary Figure S3B**).

### 3.6 Mining for differential immune cells between CR-relevant risk subgroups

**Figure 5:**
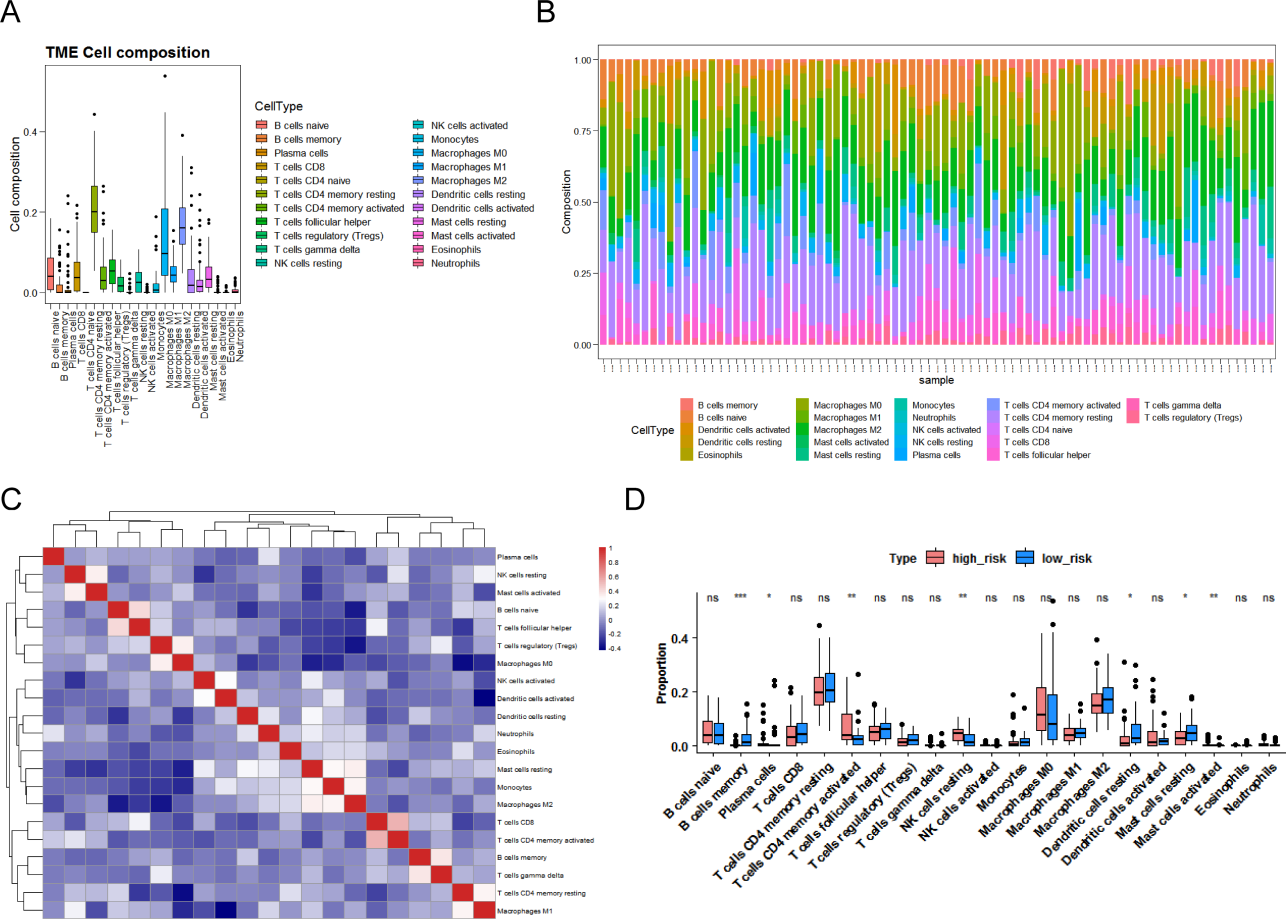
Landscape of immune infiltration in LUAD. **(A)** Box plot of the proportion of immune cells in the training set. **(B)** Bar chart of immune cell proportion stacking. **(C)** Heatmap of correlation between 22 immune cells. Red indicates a positive correlation, and blue indicates a negative correlation. **(D)** Box plots of differences in 22 immune cells in the high- and low-risk groups. *P <*0.05 indicates significance. Pink represents the low-risk group, blue represents the high-risk group, “ns” represents no significant difference, “*” represents *P <*0.05, “**” represents *P <*0.01, and “***” represents *P <*0.001.

To clarify the association between the CR-relevant risk model and immune infiltration, we recognized the differential immune cells between two CR-relevant risk subgroups. Firstly, 81 specimens (high risk = 41, low risk = 40) were incorporated to compute the fraction of each immune infiltration cell after excluding samples with *p*-value >0.05 via CIBERSORT algorithm (**Figures 5A, B**). In addition, these 22 immune cells all had negative correlations with each other (**Figure 5C**). Subsequently, according to Wilcoxon test, seven differential immune cells (memory B cells, activated mast cells, resting NK cells, activated memory CD4 T cells, resting dendritic cells, plasma cells, and resting mast cells) were authenticated between the two subgroups (**Figure 5D**).

### 3.7 The CR-relevant risk score was associated with immunotherapy response

**Figure 6:**
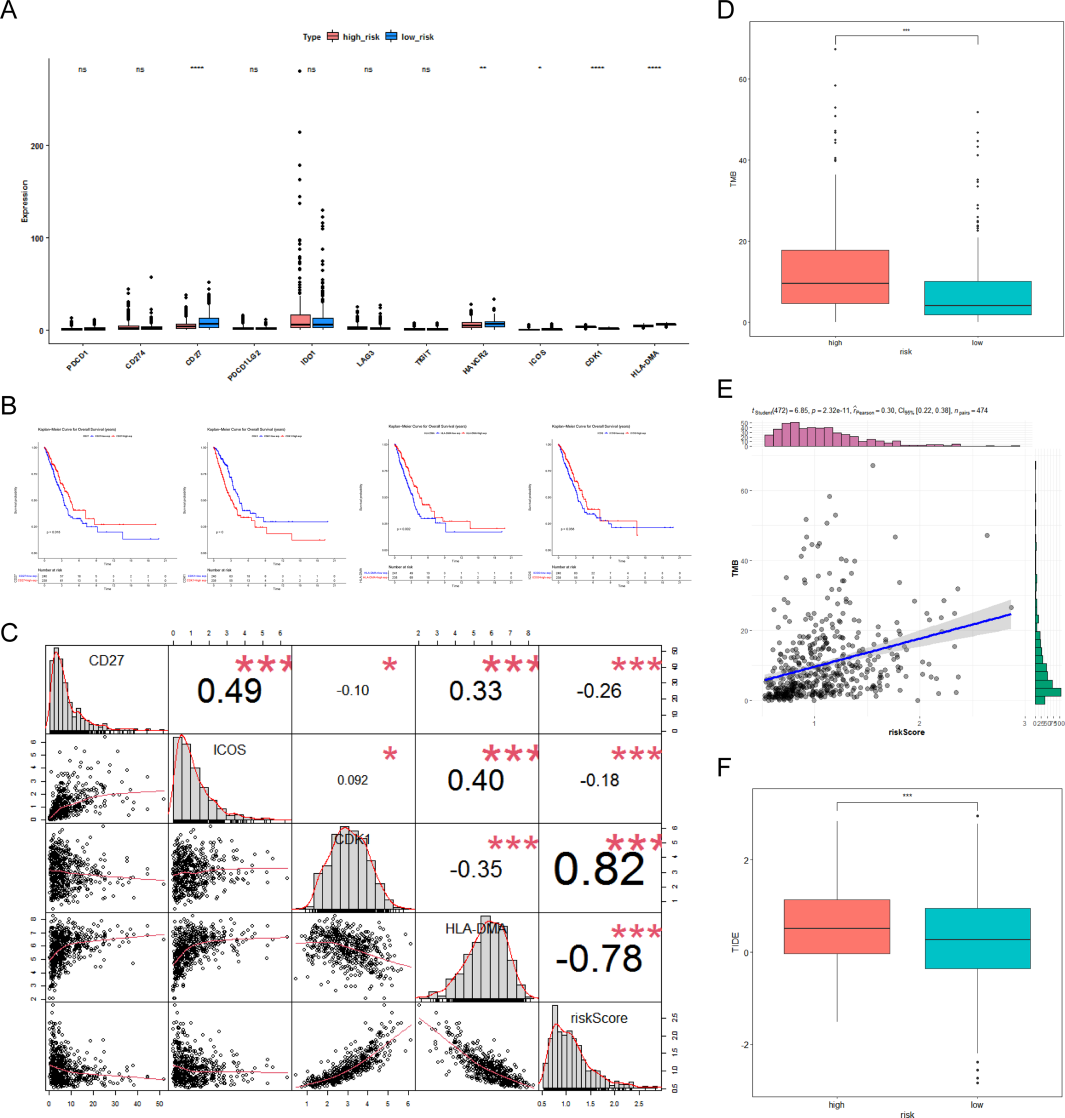
Molecular analysis of immune checkpoint and differential analysis of immunotherapy. **(A)** Box plot of immune checkpoint expression between the high- and low-risk groups. Pink represents the low-risk group, blue represents the high-risk group, “ns” represents no significant difference, “*” represents *P <*0.05, “**” represents *P <*0.01, and “****” represents *P <*0.0001. **(B)** Kaplan–Meier (K–M) survival curves for high- and low-differential immune checkpoint expression, including CD27, CDK1, HLA-DMA, and ICOS. **(C)** Correlation of immune checkpoints with risk scores. “*” represents *P <*0.05; “***” represents *P <*0.001. **(D)** Box plot of differences in tumor mutational burden (TMB) between the high- and low-risk groups. **(E)** Scatter plot of TMB correlation with risk score. “***” represents *P <*0.001. **(F)** Box plot of differences in tumor immune dysfunction and exclusion (TIDE) between the high- and low-risk groups.”***” represents *P <*0.001.

Due to the effectiveness of immunotherapy in cancer treatment, the association between risk score and immunotherapy was researched. The expression differences of five immune checkpoint molecules (CD27, HAVCR2, ICOS, CDK1, and HLA-DMA) were noticeable between the two risk score groups (**Figure 6A**). In addition, CD27, CDK1, HLA-DMA, and ICOS were notably correlated with the survival of LUAD patients and were defined as critical ICGs (**Figure 6B**). **Figure 6C** indicated that the CR-relevant risk score was notably negatively correlated with HLA-DMA and positively correlated with CDK1. Subsequently, TMB was distinctly different between the two subgroups, and it was remarkably positively associated with risk score (**Figures 6D, E**). Moreover, **Figure 6F** revealed that the high-risk group was accompanied by higher TIDE scores. In summary, the CR-relevant risk score could be utilized to estimate the effectiveness of immunotherapy in LUAD patients, and the effect was worse in the high-risk group.

### 3.8 Drug screening for LUAD

**Figure 7:**
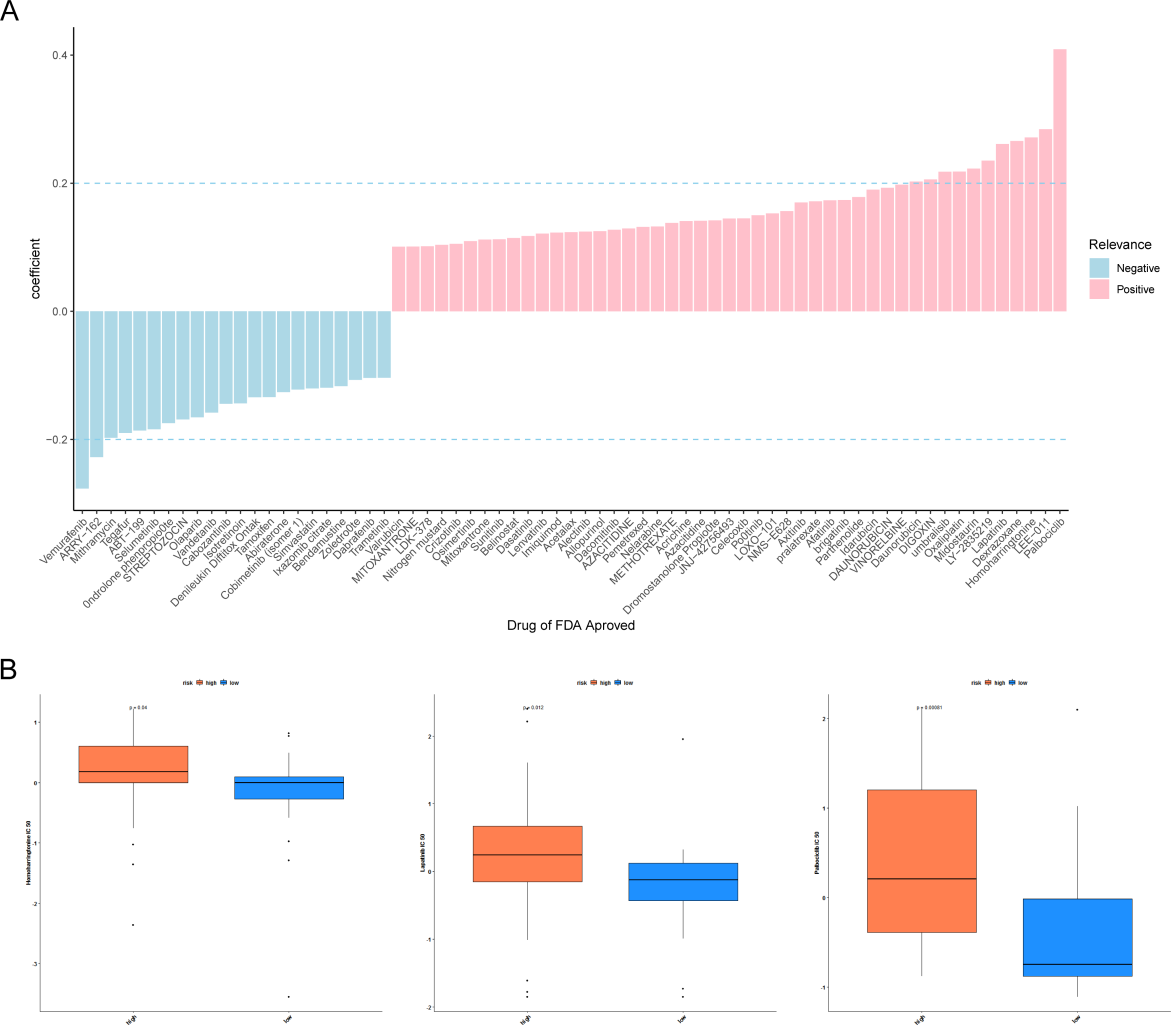
Drug sensitivity analyses. **(A)** Histogram of half-maximal inhibitory concentration (IC50) correlation with risk score. Pink indicates a positive correlation and blue indicates a negative correlation. **(B)** Differences in IC50 of homoharringtonine, lapatinib, and palbociclib across the high- and low-risk groups. *P <*0.05 indicates a significant difference.

To find effective therapeutic drugs for the two groups of patients with differing risk, we performed a drug sensitivity analysis. **Figure 7A** manifested that the risk score was remarkably positively correlated with homoharringtonine, dexrazoxane, lapatinib, LEE-011, and palbociclib, while it was remarkably negatively associated with vemurafenib and ARRY-162 (|cor| > 0.2, *p* < 0.05). In addition, the IC50 of homoharringtonine, lapatinib, and palbociclib was distinctly different between the two cell-line-risk subgroups (**Figure 7B**).

### 3.9 Discrepancies in the expression of prognostic genes

**Figure 8:**
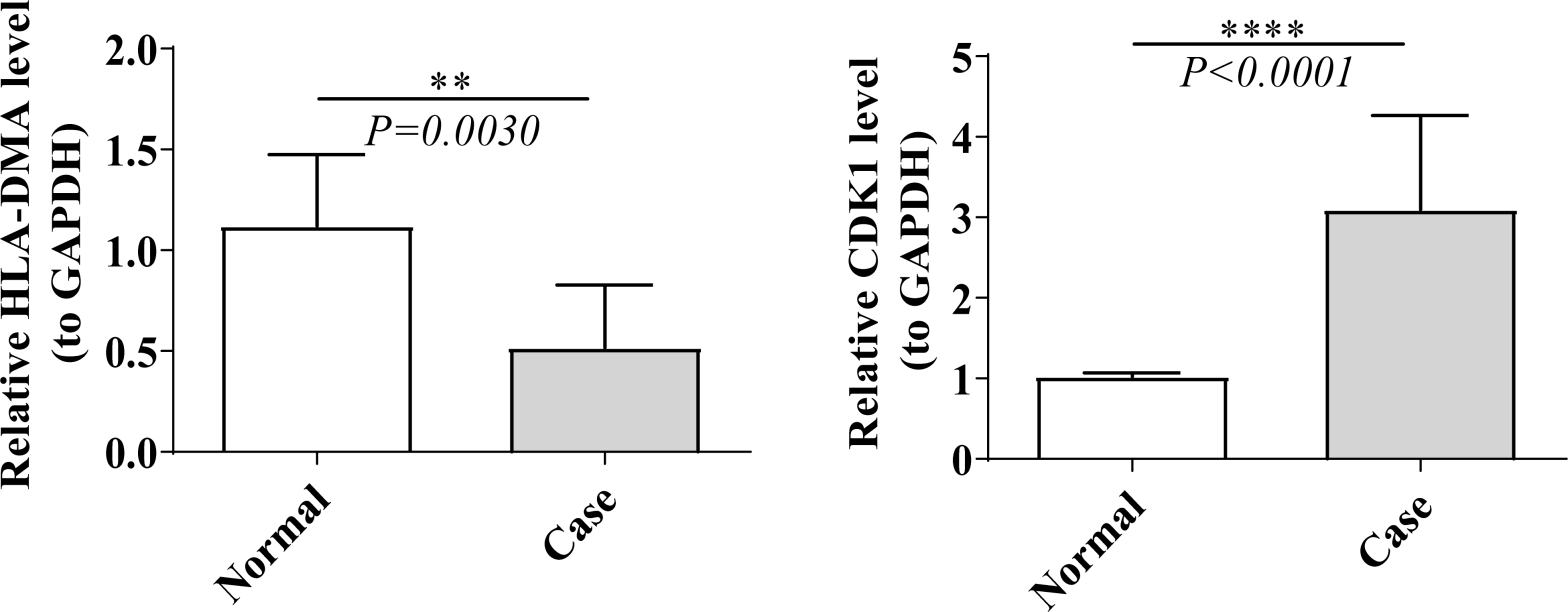
Real time-quantitative PCR (RT-qPCR) validation of HLA-DMA and CDK1. “**” represents *P* <0.01, and “****” represents *P* <0.0001.

As revealed in **Supplementary Table S2**, the expression of HLA-DMA was notably lower (log_2_FC value was negative) in LUAD specimens than in NC specimens, and the CDK1 was notably higher (log_2_FC value was positive) in LUAD specimens than in NC specimens. We then tested the expression of prognostic genes at the mRNA level in the clinical samples that we collected. Consistent with the results from the public database, the expression of CDK1 was higher in LUAD samples than in normal samples, and the expression of HLA-DMA was lower in LUAD samples (**Figure 8**).

## 4 Discussion

A vast array of biochemical processes are regulated by circadian rhythms. Circadian perturbation disrupts clock function, which increases the probability of tumorigenesis and cancer progression by influencing cell proliferation, DNA repair, metabolism, and the tumor microenvironment (TME) (40–44). In lung cancer, abnormal circadian rhythm gene expression might accelerate the initiation and progression through multiple pathways, including the regulation of c-myc, metastatic factors, immune cells, and cell cycle proteins (45). New therapeutic strategies are emerging based on the potential mechanisms of the clock interacting with cancer, generally including chronotherapy and pharmacological molecules (15). Chronotherapy optimizes dosing time to achieve optimal efficacy. Recent research has reported that CR of cancer immunosurveillance might influence tumor size and cancer immunotherapy. Immunotherapy is more effective when synchronized with dendritic cell functions, as the rhythmic transport of dendritic cells to the tumor-draining lymph node regulates the circadian response of tumor-antigen-specific CD8 T cells (46). Pharmaceutical compounds that target essential components of the circadian clock, such as GSK-3β inhibitors, CRY stabilizers, CRY inhibitors, CK1 inhibitors, CK2 inhibitors, and REV-ERB agonists, are being developed and show anti-cancer potential (47, 48). Thus, CR-related genes have the potential to be novel biomarkers to predict prognosis.

Using public data, we established and verified a CR-related risk model based on two prognosis-related genes—CDK1 and HLA-DMA—selected from 12 CR-related DEGs. The low-risk group showed a better prognosis than the high-risk group, with the AUC values of 1, 3, and 5 years all greater than 0.6. In addition, risk score and stage were identified as reliable independent predictors for LUAD through univariate and multifactorial Cox regression analyses. A nomogram consisting of risk score and stage could effectively predict the survival rate of LUAD patients. To confirm the prognostic effect of this risk model in actual clinical cases, we performed qRT-PCR analysis on the collected 10 normal and LUAD tissues. The results were consistent with the public data analysis.

CDK1 belongs to cyclin-dependent kinases (CDKs) family, a set of serine/threonine protein kinases that participate in the cell cycle process (49). Abnormal activation of CDKs could lead to excessive cell division which is a hallmark of cancer (50, 51). Several clinical trials of CDK inhibitors, including early pan-CDK inhibitors, multitarget CDK inhibitors, and selective CDK inhibitors, have been conducted in multiple malignancies, and some of them had demonstrated significant antitumor effects. The most successful CDK inhibitors are the dual CDK4/6 inhibitors, which could specifically block the retinoblastoma protein pathway participating in the transition from the G1 to the S phase of the cell cycle, thereby preventing cancer cell progression (52). Currently, CDK4/6 inhibitors have been approved by the FDA for the treatment of advanced-stage hormone-receptor-positive, HER2-negative breast cancer and also show benefits in non-small cell lung cancer, melanoma, and head and neck squamous cell carcinoma (53, 54). As the most essential cell cycle Cdk in the process of driving cell division, CDK1 initiates mitosis by binding and being activated by cyclins A and B during the late S/G2 phase (55). CDK1 is involved in multiple oncogenic pathways, and inhibiting the expression and activation of CDK1 might exert an anti-tumor effect (56). Several researchers had revealed that CDK1 inhibition induces MYC-dependent apoptosis through a synthetic lethal interaction between CDK1 and MYC in lymphomas, hepatoblastomas, and especially breast cancers (57–59). Additionally, KRAS/CDK1 interaction also exerts a robust synthetic lethal effect worthy of a further study (60). In lung cancer, CDK1 is upregulated compared with normal tissues and was negatively correlated with the overall survival of lung cancer patients (61, 62). In our research, CDK1 showed a higher expression in LUAD specimens than in normal lung tissues and was positively correlated with the CR-relevant risk score. It turns out that CDK1 is a protective factor for LUAD outcomes, which may be a prognostic and treatment biomarker in LUAD.

HLA-DMA is a protein-coding gene playing an important role in MHC class II/peptide complex formation (63) and protects empty MHC class II molecules from functional inactivation in the pathway of class II antigen presentation (64). Oldford et al. demonstrated that the upregulation of HLA-DMA might strengthen the immune response dominated by Th1 CD4 T cells and improve patient survival in breast cancer (65). Recently, HLA-DMA has been proposed to be a potential prognostic biomarker in breast cancer and glioblastoma (66, 67). A latest research observed that HLA-DMA might suppress LUAD cell proliferation by arresting the cell cycle at the S phase, and the decreased HLA-DMA expression in LUAD tissues was correlated with worse OS. The research found that HLA-DMA might alter the prognosis and immunotherapy sensitivity in LUAD by regulating the TME status (68). We obtained the same results in that HLA-DMA showed a lower expression in LUAD specimens than in normal lung tissues. In our CR-related risk model, the risk score was negatively correlated with the expression of HLA-DMA, illustrating that HLA-DMA is correlated to the poor survival outcomes of cancer patients.

Through GSVA enrichment analysis, we found 11 pathways to be associated with CR-relevant risk score, including cell cycle, cellular senescence, DNA replication, fanconi anemia pathway, homologous recombination, lysosome, mismatch repair, nucleotide excision repair, oocyte meiosis, progesterone-mediated oocyte maturation, and RNA transport. Thereinto, cell growth and development and DNA repair-relevant pathways were highly expressed in the high-risk subgroup, which indicates a worse prognosis. DNA repair is a part of DNA damage response, and defects in the DNA damage response can cause cancer predisposition. Drugs targeting the DNA damage response exhibit anti-tumor effects by exploiting synthetic lethal mechanisms (69, 70). Excessive cell growth and proliferation are a hallmark of cancer which is often caused by cell cycle disorders. Moreover, Cdk1 activity is a pivotal factor for cell cycle entry. Many studies inhibiting tumor proliferation by targeting CDK1 have obtained meaningful results—for instance, miR-181a inhibits cell proliferation by regulating the expression of CDK1 in NSCLC cells (71). CDK1 knockdown could reduce NSCLC cell proliferation, invasion, and migration by avoiding NUCKS1 overexpression (72).

Immune checkpoint inhibitors have altered the treatment of NSCLC, especially PD-1/PD-L1 immune checkpoint inhibitors. However, the issues of immune-related adverse reactions, low response rate, and drug resistance limit the clinical application of immune checkpoint inhibitors (73). Great efforts have been devoted to searching for effective tumor biomarkers to predict the treatment response and prognosis of LUAD patients. A great quantity of biomarkers discovered so far, including PD-L1 expression levels, TMB, and TME, have not been widely used clinically due to various type defects (74). Since a tumor immune microenvironment plays an important role in tumor development and immune escape (75), in this study, we found that memory B cells, resting dendritic cells, and resting mast cells showed a higher expression in the low-risk group. A reduction of these immune cells may be associated with poor prognosis. In addition, we defined four differentially expressed immune checkpoint molecules (CD27, CDK1, HLA-DMA, and ICOS) as critical ICGs. There is emerging evidence revealing the function of circadian clocks in the potential symbiotic interactions between cancer cells and TME. On one hand, cancer cell clock components impact angiogenesis, tumor-promoting inflammation, and immune evasion of TME; on the other hand, the TME clock components impact cancer cell stemness modulation, tumor growth, metastasis, and therapeutic efficacy (76). CDK1 and HLA‐DMA are contained within our risk model. A systematic analysis showed that the expression of CDK1 is related to multiple immunomodulator and chemokine expression and increased infiltration of numerous immune cells, which could reshape the tumor immune microenvironment (77). HLA‐DMA expression has been shown to be positively correlated with CD8+ T cells (68). CD27 was a co-stimulatory molecule that stimulates T-cell proliferation and differentiation to effector and memory T cells binding to its ligand CD70. Agonistic CD27 antibodies induce increased antitumor immunity and have shown clinical benefit mainly in hematological aspects (78). Like CD27, ICOS is another co-stimulatory molecule involved in adaptive T-cell responses and T-cell differentiation. It was induced by T-cell receptor engagement or CD28 co-stimulatory signaling and then expressed on CD4+ and CD8+ T cells. There was a higher expression of ICOS in tumor-infiltrating lymphocytes, especially regulatory T cells in cancer. ICOS antibodies could effectively evoke an antitumor immune response via depletion of ICOS+ Treg cells (79, 80). We believed that differentially expressed immune cells may influence tumor immunity and patient survival. The decreased number of these immune cells and immune checkpoint molecules might be related to patient survival by regulating tumor immunity.

TMB refers to the number of somatic mutations within tumors. Higher somatic TMB was previously thought to be associated with better survival in patients treated with immune checkpoint inhibitors since higher TMB promotes more production of neoantigens which could be recognized by T cells (81). Our data showed a positive correlation between TMB and risk scores, which indicated that patients in high-risk groups were more likely to benefit from immunotherapy. Furthermore, we calculated the TIDE score, which was developed to predict immune checkpoint blockade response (82). The result showed that patients in the high-risk group with higher TIDE scores were associated with a poorer response to immunotherapy and shorter survival in patients treated with anti-PD1 and anti-CTLA4. This finding was contrary to the results of the TMB analysis. TMB is a controversial biomarker. It has been reported that TMB does not correlate with PD-L1 expression under the influence of both tumor suppressor genes and oncogenes (83). Only a small proportion of neoantigens are recognized by T cells. Furthermore, factors related to individual immune microenvironment had an impact on T-cell-mediated cell killing (81). For these reasons, TMB itself was not sufficient to be a prognostic and predictive factor. Based upon the results of the studies described above, we still suppose that patients in the low-risk group might achieve better immune checkpoint inhibitor outcomes by regulating tumor immunity.

According to a drug sensitivity analysis, patients in the low-risk group were more sensitive to vemurafenib and ARRY-162. Vemurafenib is a selective BRAF kinase inhibitor that can effectively inhibit the activity of BRAF V600E. The V600E substitution in BRAF is the most common somatic mutation in melanoma, and vemurafenib was the first BRAF inhibitor approved for the treatment of late-stage BRAF V600E-positive malignant melanoma (103). Moreover, it is effective for other tumors harboring BRAF V600E mutations, including papillary thyroid cancer, hairy-cell leukemia, and NSCLC (104–106). ARRY-162 (binimetinib) is a reversible MEK1/2 inhibitor which is often used in conjunction with encorafenib in BRAF V600E-mutant metastatic melanoma, NSCLC, and colorectal cancer (107–109).

Furthermore, patients in the high-risk group were more sensitive to five anti-cancer drugs (homoharringtonine, dexrazoxane, lapatinib, LEE-011, and palbociclib), especially homoharringtonine, lapatinib, and palbociclib. Homoharringtonine, a plant alkaloid isolated from *Cephalotaxus* species, exhibits anti-tumor effects through inhibiting protein translation. Homoharringtonine is mainly used in hematological tumors, especially in tyrosine kinase inhibitor-resistant chronic myeloid leukemia. In recent studies, homoharringtonine has also shown anticancer activity in solid tumors such as breast cancer, liver cancer, and lung cancer (84–86). Lapatinib is a tyrosine kinase inhibitor targeting both EGFR and HER2 signaling. Lapatinib, in combination with capecitabine or trastuzumab, was applied in advanced HER2-positive breast cancer (87, 88). Moreover, lapatinib can improve the survival for HER2-positive breast cancer patients with brain metastases due to its ability to cross the blood–brain barrier (89). Palbociclib is the first CDK4/6 inhibitor developed. As mentioned earlier, its anti-tumor mechanism involves cell cycle arrest. The combination of palbociclib and endocrine therapy has shown better outcomes in both early-stage and metastatic hormone receptor-positive, HER2-negative breast cancer (90, 91). Circadian rhythm genes play a key role in regulating physiological processes such as cell proliferation, apoptosis, and metabolism (92) and may influence tumor progression by modulating cell-cycle-related signaling pathways. Circadian rhythm genes, such as Per2, have been demonstrated to regulate the expression of cyclin D1, which impacts the transition from the G1 phase to the S phase (93). In tumors such as lung adenocarcinoma, the binding of cyclin D1 to CDK4/6 is crucial for cell cycle progression, and palbociclib disrupts the tumor cell cycle progression by selectively inhibiting the activity of CDK4/6 (94, 95). When cyclin D1 expression is reduced, palbociclib binds more strongly to CDK4/6, resulting in a more effective inhibition of tumor cell proliferation (96). In addition, as an antitumor drug, HHT may reduce the expression and activity of cyclin D1 by downregulating the activity of the PI3K-AKT-mTOR (97) and RAS-RAF-MEK-ERK pathways (98), thus preventing the transition of the cells from the G1 phase to the S phase (99, 100). Lapatinib inhibits the activation of these pathways by targeting EGFR and HER2, thereby blocking cell cycle progression and inducing apoptosis in tumor cells (101, 102). Therefore, the three drugs (HHT, lapatinib, and Palbociclib) may collectively intervene in the regulation of the cell cycle and inhibit the proliferation of tumor cells by affecting the signaling pathways related to circadian genes, consequently producing a synergistic effect on the treatment of tumors such as lung adenocarcinoma. It is necessary to integrate driver gene status, drug sensitivity, adverse reactions, and distant metastasis to choose effective drugs.

## 5 Conclusion

This study identified prognosis-related genes in LUAD based on the data of TCGA databases and performed a simple RT-qPCR verification. In future studies, more experiments are necessary to validate the capability of the genes as reliable biomarkers. By establishing animal models to simulate the development of lung adenocarcinoma, we can further explore the impacts of drugs on the tumor microenvironment of lung adenocarcinoma under the interaction between drugs and circadian rhythm genes in order to gain a deeper understanding of the relationship between the drugs and lung adenocarcinoma. Furthermore, the function of these genes and the potential relationship with lung cancer remain to be explored. In summary, the prognostic model constructed based on circadian-rhythm-related genes provides new ideas for the improvement of prognosis and treatment of LUAD.

## Data Availability

All extracted data from the TCGA database (https://www.cancer.gov/ccg/research/genome-sequencing/tcga), the GEO database (http://www.ncbi.nlm.nih.gov/geo) and GSEA-MsigDB database (https://www.gsea-msigdb.org/gsea) used and/or analyzed are available from the corresponding author upon reasonable request.

## Supplementary material

Supplementary Figure 1 Differences between cluster 1 and cluster 2 for different subtypes of clinical features, including M stage, N stage, Stage, T stage, and gender.

Supplementary Figure 2 Correlation analysis between risk model and clinical factors. **(A)** Box plots of differences in risk scores between different clinical subtypes. “NS” represents no significance, “*” represents *P <*0.05, and “**” represents *P <*0.01. **(B)** Kaplan–Meier (K–M) survival curves in patients with different clinical subtypes. *P <*0.05 indicates significance.

Supplementary Figure 3 Gene set variation analysis (GSVA) analysis. **(A)** Heatmap of correlation between KEGG pathways and risk scores. **(B)** Heatmap of differential pathway enrichment expression in the high- and low-risk groups.
