# Supplementary figures and images for: A prognostic model of lung adenocarcinoma constructed based on circadian rhythm genes and its potential clinical significance
Source: Front Oncol. 2025 Feb 18;15:1464578. doi: 10.3389/fonc.2025.1464578 (PMC11876053; doi:10.3389/fonc.2025.1464578)

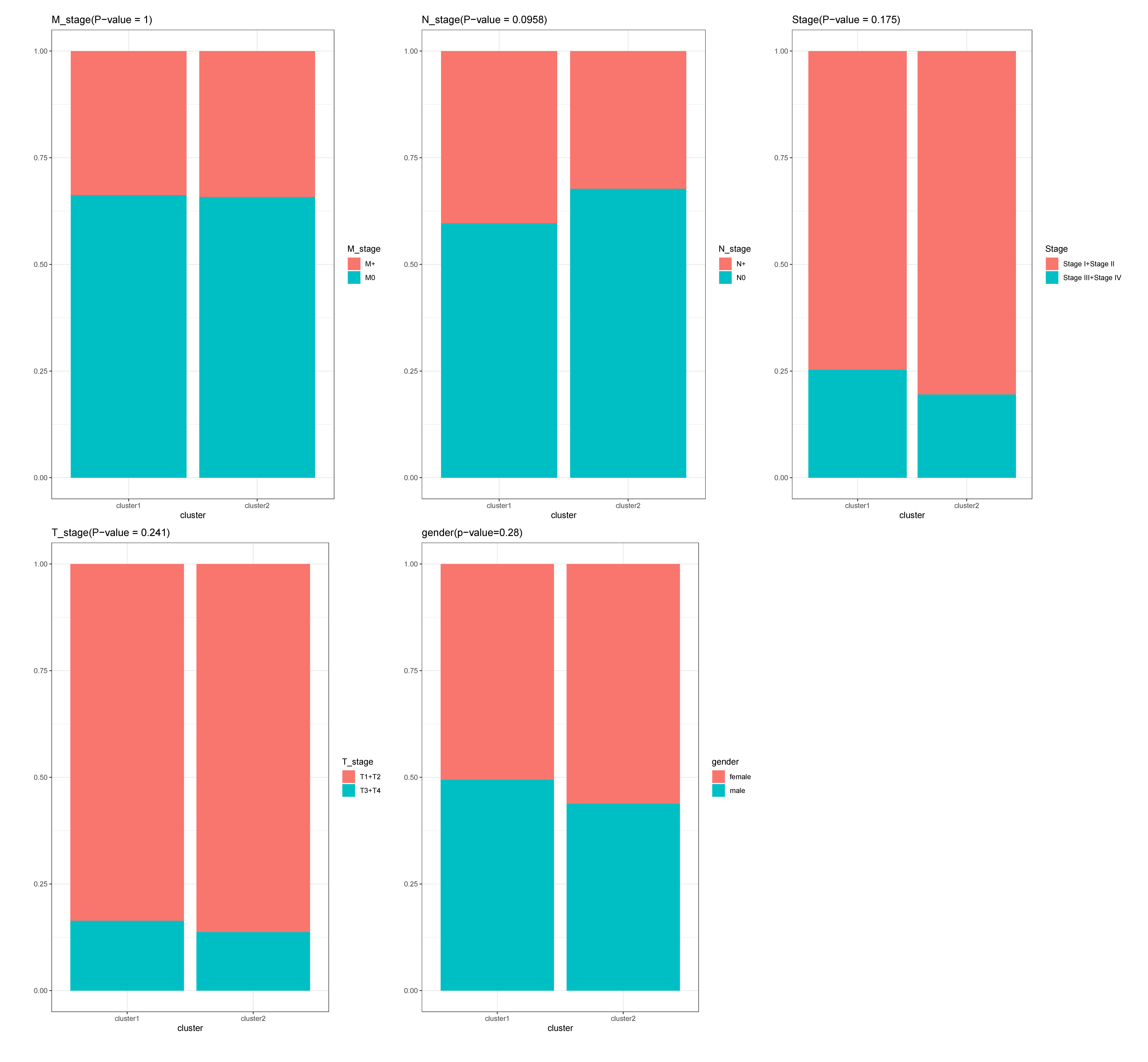

Supplement: Supplementary Figure 1 — Differences between cluster 1 and cluster 2 for different subtypes of clinical features, including M stage, N stage, Stage, T stage, and gender. [file Image1.tif]

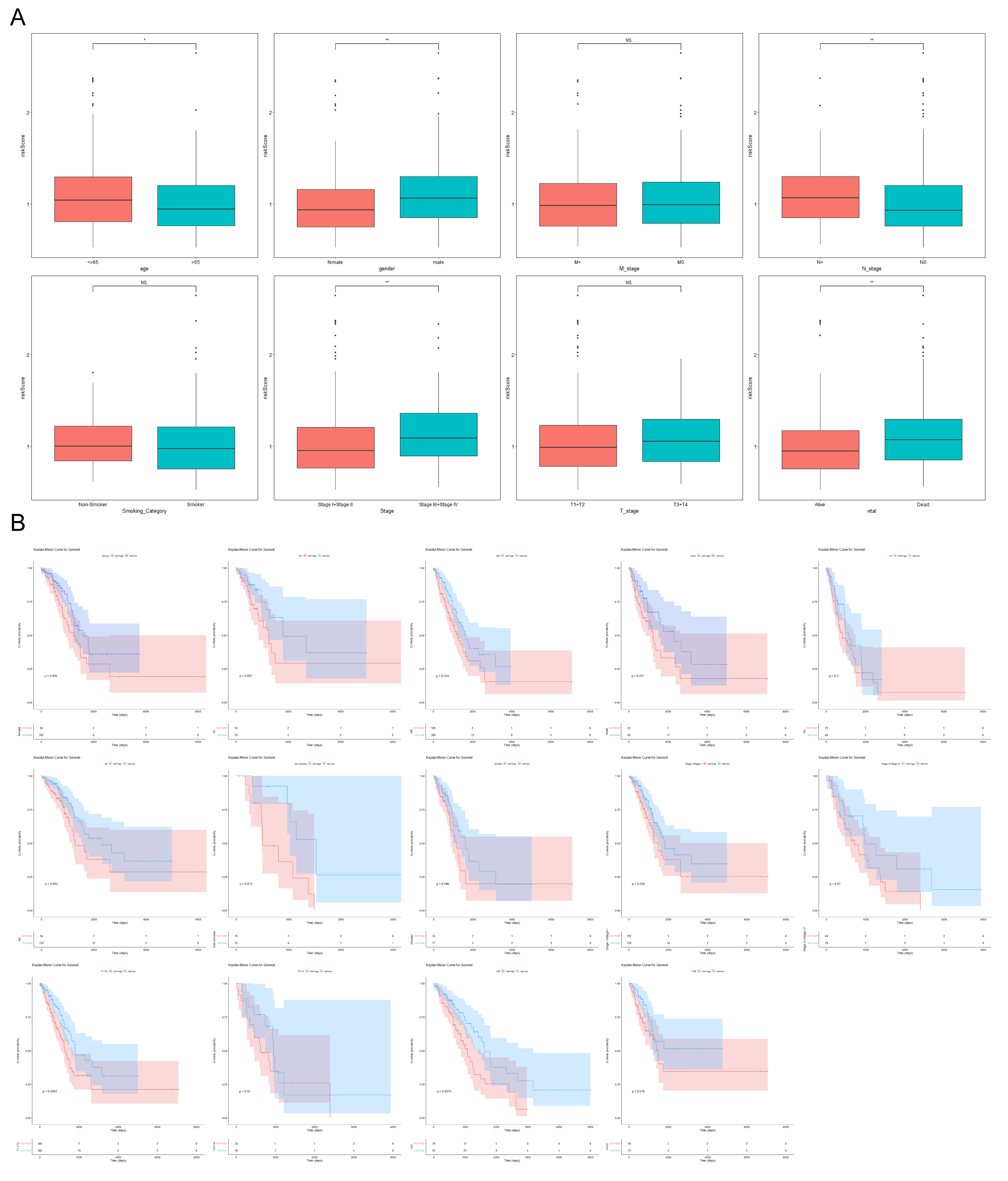

Supplement: Supplementary Figure 2 — Correlation analysis between risk model and clinical factors. (A) Box plots of differences in risk scores between different clinical subtypes. “NS” represents no significance, “*” represents P <0.05, and “**” represents P <0.01. (B) Kaplan–Meier (K–M) survival curves in patients with different clinical subtypes. P <0.05 indicates significance. [file Image2.tif]

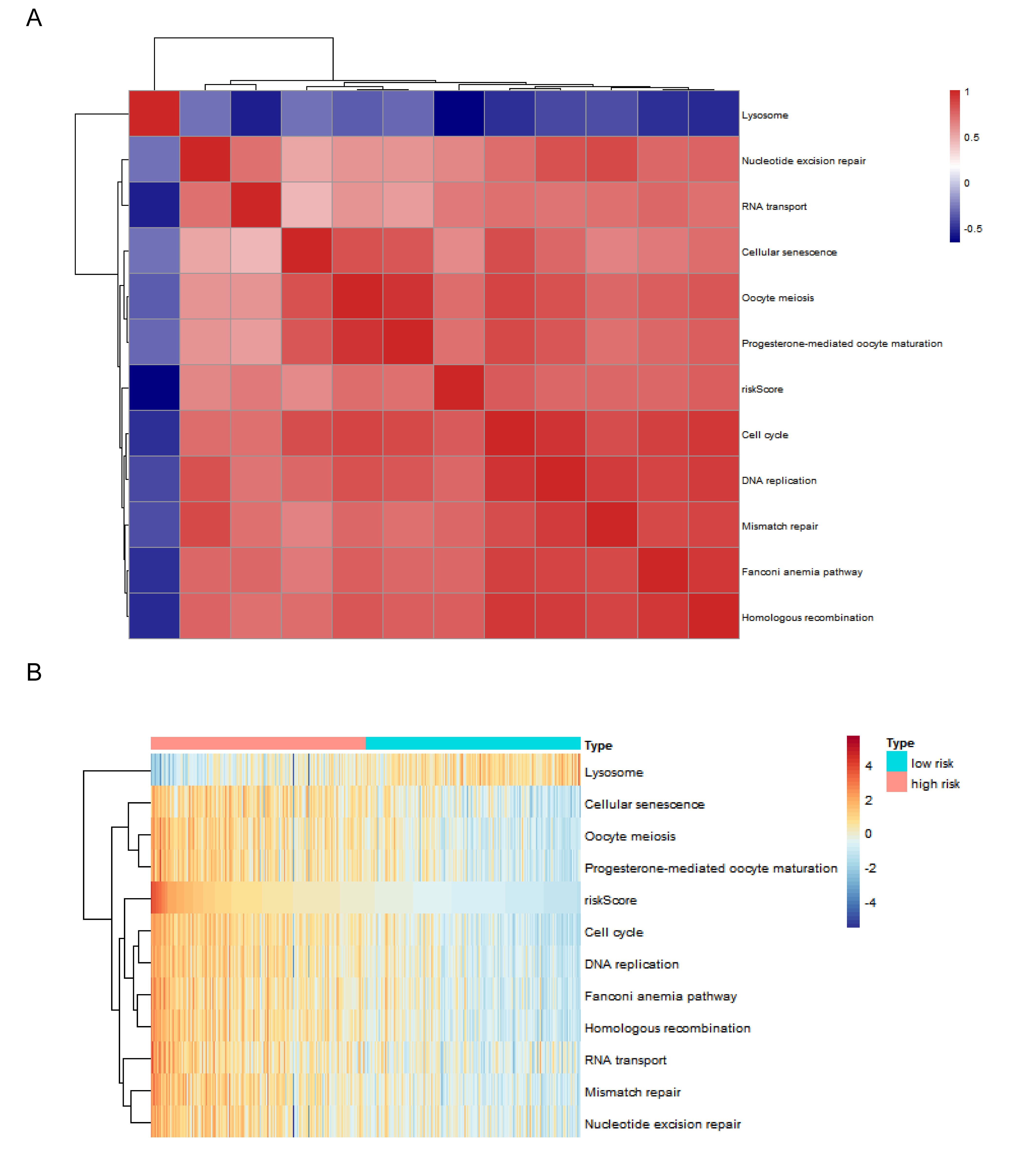

Supplement: Supplementary Figure 3 — Gene set variation analysis (GSVA) analysis. (A) Heatmap of correlation between KEGG pathways and risk scores. (B) Heatmap of differential pathway enrichment expression in the high- and low-risk groups. [file Image3.tif]
